# Optimization and Synthesis of Nano-Niosomes for Encapsulation of Triacontanol by Box–Behnken Design

**DOI:** 10.3390/molecules29184421

**Published:** 2024-09-18

**Authors:** Alfredo Amaury Bautista Solano, Gloria Dávila-Ortiz, María de Jesús Perea-Flores, Alma Leticia Martínez-Ayala

**Affiliations:** 1 Departamento de Biotecnología, Centro de Desarrollo de Productos Bióticos, Instituto Politécnico Nacional (IPN), Carretera Yautepec-Jojutla s/n-Km 85, San Isidro, Yautepec 62739, Morelos, Mexico; abautistas2103@alumno.ipn.mx; 2 Departamento de Ingeniería Bioquímica, Escuela Nacional de Ciencias Biológicas, Instituto Politécnico Nacional (IPN), Av. Wilfrido Massieu Esq. Miguel Stampa s/n, Zacatenco, Alcaldía Gustavo A. Madero, Ciudad de Mexico 07728, Mexico; gdavilao@yahoo.com; 3 Centro de Nanociencias y Micro y Nanotecnologías, Instituto Politécnico Nacional (IPN), Av. Luis Enrique Erro s/n, Unidad Profesional Adolfo López Mateos, Zacatenco, Alcaldía Gustavo A. Madero, Ciudad de Mexico 07738, Mexico; mpereaf@ipn.mx

**Keywords:** niosomes, triacontanol, policosanol, nanotechnology, encapsulation

## Abstract

Triacontanol is a long-chain primary alcohol derived from policosanol, known for its diverse biological activities, including functioning as a plant growth regulator and exhibiting anti-inflammatory and antitumoral effects. However, its application is limited due to its high hydrophobicity, resulting in poor absorption and reduced therapeutic effectiveness. A potential solution to this problem is the use of niosomes. Niosomes are carriers composed of non-ionic surfactants, cholesterol, charge-inducing agents, and a hydration medium. They are effective in encapsulating drugs, improving their solubility and bioavailability. The objective of this study was to optimize and synthesize nano-niosomes for the encapsulation of triacontanol. Niosomes were synthesized using a thin-film hydration method combined with ultrasonication, following a Box–Behnken design. Niosomes were characterized using various techniques including dynamic light scattering, Fourier-transform infrared spectroscopy (FTIR), confocal microscopy, high-resolution scanning electron microscopy, and transmission electron microscopy (TEM). Formulation 14 of niosomes achieved the desired size, polydispersity index (0.198 ± 0.008), and zeta potential (−31.28 ± 1.21). FTIR analysis revealed a characteristic signal in the 3400–300 cm^−1^ range, indicating intermolecular interactions due to a bifurcated hydrogen bond between cholesterol and S60. Confocal microscopy confirmed the presence of triacontanol through Nile Red fluorescence. TEM revealed the spherical structure of niosomes.

## 1. Introduction

Triacontanol C_30_H_62_O is a saturated primary fatty alcohol derived from policosanol. Policosanol is a mixture of long-chain, high-molecular-weight alcohols characterized by having 22 to 34 carbon atoms [1,2,3]. Triacontanol exhibits different biological activities. It functions as a dietary supplement and lipid-lowering agent as well as an antitumor agent, by altering DNA synthesis, facilitating transmembrane permeation, and regulating matrix metalloproteins, and vascular endothelial growth factor [4]. Triacontanol regulates the genetic expression of plants under stress conditions. Its mechanisms involve antioxidant defense systems of enzymatic and non-enzymatic nature. Triacontanol is also involved in producing photosynthetic pigments, thus increasing plant biomass and transpiration rate, soluble sugars, proteins, free amino acids, nutrient absorption, nitrogen fixation, and the synthesis of essential oils and bioactive compounds. Generally, triacontanol is considered a plant growth promoter. However, triacontanol application is limited due to its highly hydrophobic molecular structure, which makes its use difficult [5,6,7].

One solution is the use of nanotechnology. Nanotechnology is one of the most promising areas of knowledge, where disciplines such as medicine, chemistry, biology, and engineering converge. Among nanotechnology applications is the design, synthesis, and characterization of nanomaterials. Nanomaterials are systems at the nanometer scale (1–100 nm) [8]. Nanomaterials have become a source of various applications, such as the development of sensors, drugs, batteries, antimicrobials, and nanocarriers [9]. Drug nanocarriers are those that can precisely guide a beneficial product to a target location without interference. A transporter must be efficient and highly biocompatible with the molecule to be transported [10].

There are different classes of nanocarriers according to their chemical composition and function. Inorganic nanocarriers are those that contain mostly inorganic elements; on the other hand, we find organic nanocarriers such as polymeric and lipid nanocarriers [11,12]. Niosomes fall within the lipid nanocarriers. Niosomes are vesicular carriers that are useful in controlled, sustained, and targeted drug delivery [13,14]. Niosomes comprise nonionic surfactant, cholesterol, charge-inducing molecules, and a hydration medium [15]. These nanocarriers have a structure consisting of two domains, one lipophilic and one hydrophilic, and they are useful in administering molecules of different solubility [16]. Niosomes are osmotically stable and protect the encapsulated molecule from metabolism and degradation, increasing therapeutic efficacy. They are also more efficient compared to other transporters, such as liposomes. Niosomes are formed by non-ionic surfactants, providing greater physicochemical stability against fusion, aggregation, and oxidation, in addition to avoiding contact with cell membranes. On the other hand, liposomes made up of phospholipids can impart toxicity to normal tissues and elicit immune response increasing the possibility of cell damage [17,18]. These advantages are only possible with niosomes on a nanometric scale, and thus it is necessary to optimize a formulation to obtain niosomes with the desired characteristics.

There are various methods to optimize formulations. One of the most efficient is the Box–Behnken response surface design. Response surface methods are mathematical techniques aimed at statistical analysis, where a response of interest is influenced by several variables to optimize a response, providing an appropriate approximation of the functional relationship among the set of independent variables with a first-order approximation function. The objective of these methods is to determine the system’s optimal operating conditions that satisfy the requirements for the niosome design of these operations and the subsequent statistical analysis for each evaluated variable [19]. The objective of this study was to design a nanometric stable niosome formulation using Box–Behnken response surface design.

## 2. Results and Discussion

### 2.1. Effect of Independent Variables on the Characteristics of Niosomes

A total of 15 different formulations of niosomes were obtained as shown in Table 1 Response surface graphs were obtained to represent the effects of the preset factors in immediate responses.

### 2.2. Effect of Independent Variables on Vesicle Size

An ANOVA analysis of the 15 formulations was performed. The effect of independent variables that may influence size was analyzed. The ANOVA analysis indicated that sonication time only affects particle size (0.0011 < 0.05). The smallest mean vesicle size was observed in formulation 14 (139.54 ± 0.93 nm) while the maximum vesicle size was found in formulation 6 (550.34 ± 6.68 nm). The effect of independent variables on the size of particles is presented in three-dimensional graphs, where the combination of two independent variables on niosome size is observed.

Niosomes contain a hydrophilic domain encapsulated by a surfactant-formed membrane, which must resist the Laplace pressure stress and not collapse. This condition is achieved by obtaining niosomes on a nanometric scale in a range of 10–100 nm. Moreover, the niosome bilayer formed by nonionic surfactants interferes with this process and prevents coalescence. However, Mashal et al., 2023, synthesized niosomes based on cationic polymers and found that this phenomenon occurs as time goes by, regardless of the niosome wall material, indicating that one way to avoid it is through a decrease in temperature [20].

**Figure 1:**
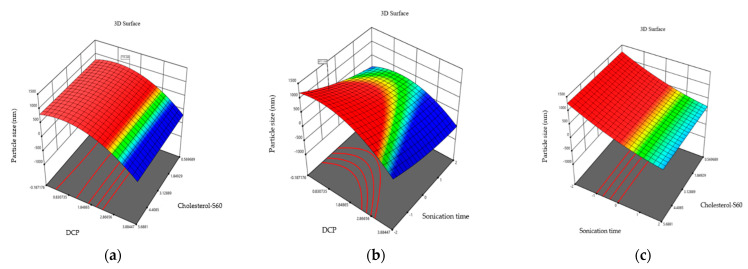
Three-dimensional response plots displaying effects on particle size. Dihexadecyl phosphate and relationship of cholesterol and S60 effect on particle size (**a**), sonication time and dihexadecyl phosphate effect on particle size (**b**), and sonication time and relationship of cholesterol and S60 effect on the particle size (**c**).

Particle size is a crucial point for niosome formulation providing them with various characteristics intended in the niosome. The graphs in Figure 1 show the effect of independent variables on particle size. It is observed that the combination of dihexadecyl phosphate (DCP) and S60-cholesterol in different molar ratios does not influence niosome size. On the other hand, sonication time affects the size of niosomes. A longer sonication time results in a reduction in size, which is consistent with the research by Nowroozi et al., 2018. They synthesized niosomes using different types of surfactants and size reduction methods, including ultrasonication and extrusion [21]. Ultrasonication results in size decrease. This effect is possibly due to the amplitude of the ultrasound used, which is the parameter that produces this effect. As amplitude increases, size decreases. Amplitude determines the amount of energy held by the sound wave [22]. It also indicates that the hydrophilic–lipophilic balance (HLB) of the surfactant affects niosome size.

HLB is a scale measure that indicates how hydrophilic and lipophilic a molecule is and its ability to decrease the surface tension between two non-miscible solvents. The HLB scale takes the hydrophilic molecular mass of the molecule and the total molecular mass of the molecule. HLB is important in determining the size of niosomes; with high HLB values, the size of niosomes will also increase, so they are directly proportional. In this study, S60 was used, which is a non-ionic surfactant belonging to the Span family. Spans are esters of sorbitan fatty acids. Sorbitan is a heterocyclic ring derived from sorbitol dehydration. Sorbitol anhydrides react with fatty acids through Fisher esterification to form Spans. S60 has an HLB value of 6, making it an ideal value for niosome formation [23]. It will be difficult for surfactants with HLB greater than 6 to form niosomes. Kulkarini et al., 2019, indicate that the surfactant used for niosome synthesis can affect their size; an example of this is sorbitan monolaurate (S20), which contains lauric acid, raising its HLB to 8, resulting in the formation of multilamellar niosomes. Moreover, the HLB value also influences the phase transition temperature of niosomes, affecting membrane fluidity, permeability, and stability. In the case of the non-ionic surfactant used, temperatures of 60–70 °C are recommended for optimal phase transition and niosome formation [24].

The size of niosomes can also be influenced by DCP, as the charger inducer can modify their size. According to Varaporn et al., 2009, the charge inducer ratio can reduce size, rupture membranes, and cause the formation of pores, leading to a faster release rate of the encapsulated molecule or even preventing niosome formation [25]. Studies on dihexadecyl phosphate reported that this charge inducer increases the size of niosomes due to niosome mechanisms of formation during the hydration process. When these particles are hydrated, certain types of charge develop in both the niosome and the encapsulated molecules. The non-ionic surfactant forms curves and divides forming closed vesicles with the objective of reducing its free energy; it is likely that the types of charge developed within this membrane modify not only the stiffness but also the rate of curvature, division, and formation of vesicles, which in turn will determine the size and polydispersity of niosomes [26].

Ghorbanizamani et al., 2021, indicate that the stabilizer also influences the size of niosomes. Cholesterol improves the hydrophobicity of the bilayer, which results in a decrease in surface free energy and therefore a decrease in particle size. However, the cholesterol in these formulations is a factor more correlated to niosome permeability and stability due to possible intermolecular interactions that may be present in conjunction with the nonionic surfactant [27].

### 2.3. Effect of Independent Variables on Polydispersity

**Figure 2:**
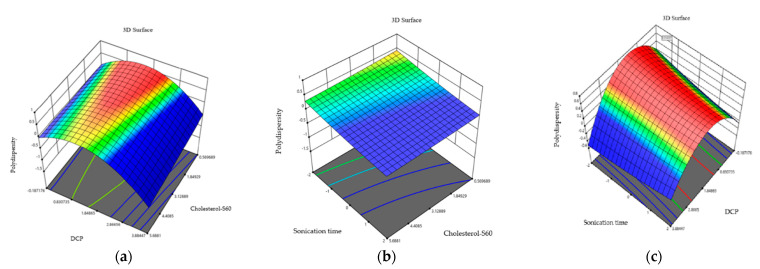
Three-dimensional response plots displaying effects on polydispersity. Dihexadecyl phosphate and relationship of cholesterol and S60 effect on polydispersity (**a**), sonication time and relationship of cholesterol and S60 effect on polydispersity (**b**), and sonication time and dihexadecyl phosphate effect on polydispersity (**c**).

The polydispersity results in the niosome formulations indicate that the highest values of polydispersity are found in formulation 5 (0.502 ± 0.303) while the lowest values are found in formulation 11 (0.124 ± 0.014). The ANOVA analysis indicates that independent variables do not have a significant effect on niosome polydispersity (0.22 > 0.05). This effect is observed in the three-dimensional graphs (Figure 2) where the independent variables are combined in the polydispersity response variable.

Formulations made of niosomes indicate PDI values of 0.124 ± 0.448. A PDI value of 1 indicates a large variation in particle size, while a value of 0 indicates no variations within the system. El-Ridy et al., 2015, reported that niosomes with a PDI lower than 0.3 are considered homogeneous systems. After the study of independent variables in formulations, we can say that dihexadecyl phosphate between the intervals of 0 and 0.5 mM maintains an ideal polydispersity for niosomes, where monodisperse colloidal systems are obtained (PDI < 0.3) [28]. However, by increasing the amount of the charge inducer, we can see that highly polydisperse niosomes are obtained where PDI > 0.5.

The graphs show that at a lower cholesterol–S60 ratio and sonication time, niosomes with monomodal dispersion can be obtained. These results coincide with Li in 2019 who synthesized niosomes using the thin-film hydration method and non-ionic surfactant in conjunction with ultrasonication for a reduction in niosome size, further evaluating variables such as the volume of the hydration medium, the co-surfactant ratio, sonication, volume, and hydration time. It is indicated that sonication reduces the size and polydispersity index, obtaining more homogeneous niosomes [26,29]. However, it can be seen how the cholesterol–S60 ratio and sonication time are obtained in a lysed graph where curvature is not present. This contrasts with research conducted by Essa in 2010, who synthesized niosomes using sorbitan monopalmitate as a non-ionic surfactant with cholesterol. It is noted that cholesterol affects both the size of niosomes and the polydispersity index. This stabilizing agent causes an increase in niosomes size and a decrease in polydispersity [30].

### 2.4. Effect of Independent Variables on Zeta Potential

**Figure 3:**
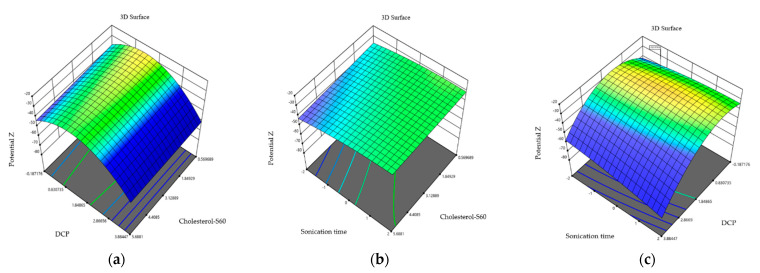
Three-dimensional response plots displaying effects on polydispersity. Dihexadecyl phosphate and relationship of cholesterol and S60 effect on zeta potential (**a**), sonication time and relationship of cholesterol and S60 on zeta potential (**b**), and sonication time and relationship of cholesterol and S60 effect on zeta potential (**c**).

The lowest zeta potential values were obtained in formulation 12 (−27.2 ± 0.21 mV) and the highest zeta potential values were obtained in formulation 4 (−40.96 ± 1.36 mV). On the other hand, the ANOVA analysis indicates that the 15 formulations of niosomes showed no significant differences for the zeta potential (0.400 > 0.05). This can be seen the three-dimensional graphs (Figure 3) where the combination of cholesterol–S60 and dihexadecyl phosphate; sonication time and cholesterol–S60; and sonication time–dihexadecyl Phosphate does not significantly influence the zeta potential of niosomes.

The zeta potential is an indicator of the particle charge through the net charge found on the surface of the particle. Particles dispersed in an aqueous system have a charge on the surface affecting the distribution of ions found in the interfacial region surrounding the particles, thus increasing the concentration of opposite ions in the surface area [31].

In the case of the zeta potential, the cholesterol–S60 ratio with the charge inducer is shown in the graphs at intervals between 3.12 and 4 mM, yielding a zeta potential of −30 mV. This corresponds to niosomes with an ideal surface charge to prevent fusion between the nanoparticles through electrostatic repulsion. Essa in 2010 inferred that both cholesterol and surfactants of the sorbitan family can contribute with a negative charge on the surface due to their hydroxyl groups. Cholesterol contains a hydroxyl group at the C-3 position; on the other hand, sorbitan monostearate contains different hydroxyl groups in the heterocyclic ring, providing charges of −11.6 ± 2.4 mV. The graph shows that shorter sonication times and higher cholesterol–S60 ratios, results in more stable niosomes [32]. This is also observed in the graph relating DCP to sonication time, where stable niosomes are obtained at charge inducer concentrations of 0.80 and 1.84 mM. Studies indicate that these zeta potential values or even higher, are due to the addition of charge inducers on the niosome surface, with values reaching as high as −50.7 when specifically using dihexadecyl phosphate; this surface charge is acquired due to the ionization of HPO_4_ groups contained in dihexadecyl phosphate where sonication times of less than 2 min also have influence [31].

Hasan in 2014 synthesized niosomes using the same type of surfactant in the absence of the charge inducer, obtaining a zeta potential around −17.1 mV, indicating moderately stable niosomes [33]. Therefore, it can be inferred that the zeta potential obtained may be largely due to the presence of the charge inductor used. Hnin in 2022 synthesized niosomes using sorbitan monostearate, cholesterol, and dihexadecyl in their formulations for cyclodextrin encapsulation. Their studies indicate that the addition of dihexadecyl phosphate is of vital importance in niosomes, improving membrane physical stability and providing steric stabilization; their research concluded that dihexadecyl phosphate increases stability as it restricts the niosome dispersions, preventing coalescence and aggregation when stored, in addition to improving encapsulation efficiency [34]. On the other hand, Priyadarshi and Abdelkader in 2011 revealed that the selected charge inducer must be in accordance with both the selected surfactant and the stabilizer due to the functional groups that can be found in these components [35].

### 2.5. Optimization

Through the Design expert software, the point prediction method was applied with the aim of obtaining the minimum particle value, maximum values of zeta potential, and minimum value of polydispersity. It was found that formulation 14, with a molar ratio of 11.4:11.4 mM cholesterol–S60, 0.6 mM dihexadecyl phosphate, and a 5 min sonication time, allowed to obtain niosomes of 139.54 ± 0.93 nm with a polydispersity of 0.198 ± 0.008 and a zeta potential of −31.28 ± 1.21 mV. The Box–Behnken design concluded that at longer sonication times and with the indicated molar ratios, niosomes in a nanometric size are obtained. With this formulation, nano-niosomes were obtained. In contrast, the polydispersity index (PDI) was less than <0.5, indicating that the sample was considered a homogeneous. The zeta potential of this formulation showed that the obtained niosomes are highly stable.

### 2.6. FTIR

The following spectra of reagents that form niosomes were obtained. The spectra show specific bands for each one of the reagents (Table 2). In the same way, the spectra of blank niosomes and niosomes with encapsulated triacontanol were obtained.

**Figure 4:**
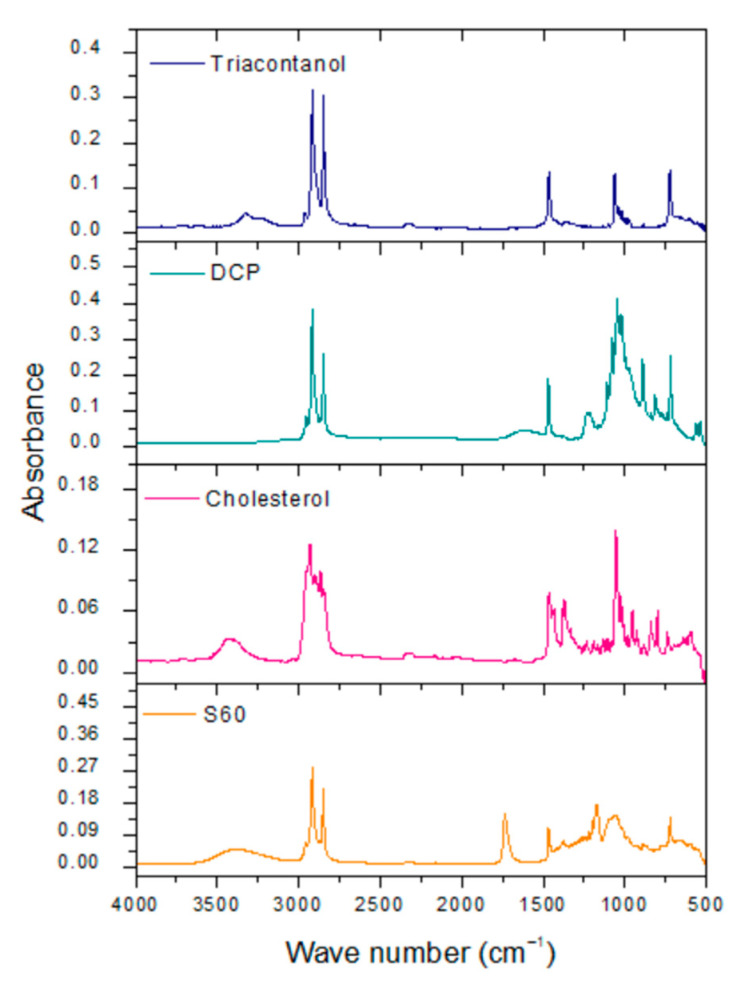
FTIR spectra of reagents for niosome formulation.

Sorbitan monostearate is a surfactant formed by a ring of sorbitol and stearic acid. The main signals obtained were at 3400–3200 cm^−1^ of the O-H bond. Another characteristic band of S60 is at 1120 cm^−1^, belonging to R-O-R dialkyl ether present in the sorbitan ring. At 1100 cm^−1^, there is the C-O tension band due to the presence of secondary alcohols in the sorbitan ring. Another reagent that forms the structure of niosomes is cholesterol. This reagent exhibits characteristics bands in the infrared spectrum such as O-H tension at 3400–3200 cm^−1^. This structure also shows a band at 3100 cm^−1^, corresponding to the unsaturation between C-5 and C-6 of carbocycle B. Dihexadecyl phosphate is the charge-inducing molecule in the niosome structure. This molecule presents phosphate groups (PO_4_^3−^) with tension vibrations at 1050–1000 cm^−1^. Triacontanol has an O-H tension band of 3400 to 3200 cm^−1^ and a C-O tension band of 1062 cm^−1^, corresponding to a primary alcohol (Figure 4) [36].

**Figure 5:**
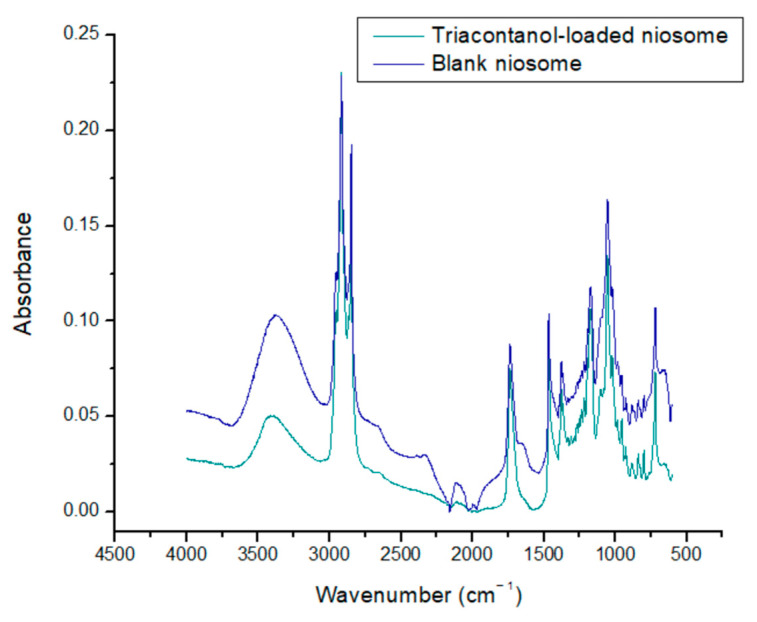
FTIR spectra of blank niosomes and triacontanol-loaded niosomes.

The spectra of blank niosomes and niosomes with encapsulated triacontanol reveal characteristic signals associated with Span 60 and cholesterol in the range of 3500–3200 cm^−1^. This signal may be broader due to the intermolecular interaction generated by a hydrogen bond between S60 and cholesterol. A characteristic signal of stretching and flexion of the hydroxyl group is located at 3445 cm^−1^, and there is a symmetrical signal at 2935 cm^−1^, corresponding to the methyl (CH_3_) cholesterol groups. The presence of a characteristic C-O signal in the range of 1675–1735 cm^−1^, corresponding to the ester bond, is reported. The signal at 1453 cm^−1^ corresponds to the symmetrical stretching of the carbonyl group of S60. Niosomes may also exhibit additional signals in the range of 1056–1173 cm^−1^, corresponding to the carbinol (C-OH) group possessed by both S60 and cholesterol. The absence of covalent signals between niosome components and triacontanol indicates that niosomes maintain their integrity as nanocarriers. The FTIR spectrum of triacontanol shows a characteristic C-O tension band, which allows researchers to distinguish between primary, secondary, and tertiary alcohols. The band is in the 1050–1150 cm^−1^ range, corresponding to the triacontanol primary alcohol (Figure 5) [37].

### 2.7. Laser Scanning Confocal Microscopy

**Figure 6:**
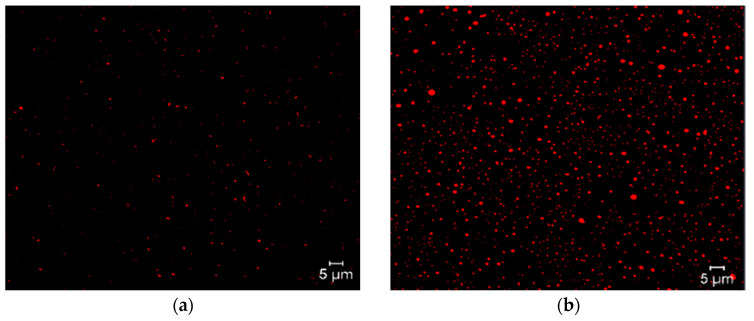
Micrographs of formulation 14 of niosomes with Nile Red fluorochrome. Blank niosomes (**a**) and niosomes with encapsulated triacontanol (**b**).

Micrographs of the different niosome formulations were obtained using Nile Red as the coloring agent, because it is highly attuned to lipophilic molecules. Micrographs reveal the formation of niosomes and the encapsulation of triacontanol, because highly lipophilic triacontanol can interact with Nile Red and produce the phenomenon of fluorescence (Figure 6).

### 2.8. Scanning Electron Microscopy

**Figure 7:**
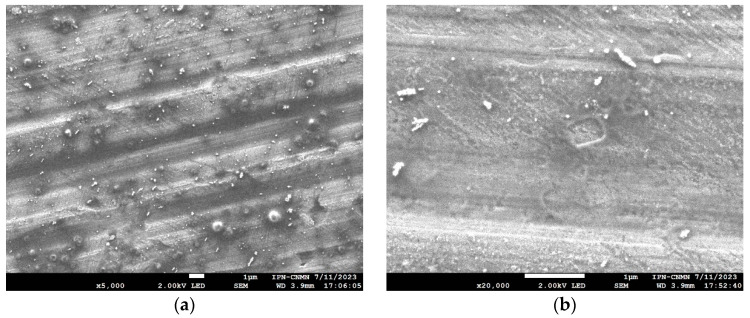
SEM micrograph of formulation 14 of niosomes. Blank niosomes (**a**) and niosomes with encapsulated triacontanol (**b**).

SEM micrographs were obtained from formulation 14 of blank niosomes and niosomes with encapsulated triacontanol (Figure 7). The niosomes found showed hemispherical and concentric structures in both the control and formulation 14 of niosomes with and without triacontanol. Micrographs reveal a smooth surface with some irregularities in shape, which may be attributed to the sample preparation process. Another possible explanation is their susceptibility to temperature. Temperature can affect niosomes; temperatures close to the phase transition of niosomes cause a significant morphological change in the vesicles.

The change in niosome shape can be related to the phenomenon known as Ostwald ripening. Ostwald ripening is a thermodynamic phenomenon that occurs when two drops of oil of different sizes come into contact in an oil/water emulsion where the fluid moves from higher to lower pressure. Ostwald ripening is explained by Laplace’s equation which indicates that the pressure experienced by droplet molecules differs from the pressure of the surrounding liquid by specific magnitude. This pressure is directly proportional to the interfacial tension of the droplet and inversely proportional to its radius. This phenomenon occurs in colloidal systems where small particles dissolved in a liquid phase basically dissolve and migrate to larger particles, as illustrated in Figure 7b [38].

### 2.9. Transmission Electron Microscopy

**Figure 8:**
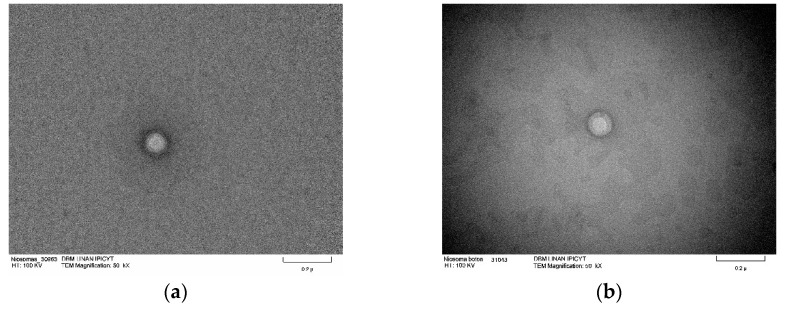

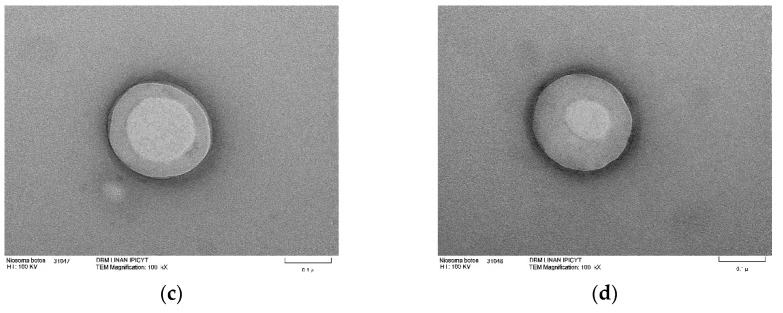
TEM micrographs of blank niosomes and niosomes with encapsulated triacontanol. Blank niosomes ((**a**,**b**); scale bar 0.2 μ) and niosomes with encapsulated triacontanol ((**c**,**d**); Scale bar 0.1 μ).

Transmission electron microscopy was used to study the niosome ultrastructure of formulation 14 (Figure 8) in the presence and absence of triacontanol. Micrographs show niosomes with a spherical shape and the size matches that obtained by dynamic light scattering. There are structures with two compartments; the aqueous compartment is inside, surrounded by the S60 association, cholesterol, and DCP. Micrographs also reveal an increase in niosomes size. In Figure 8c,d, where triacontanol is incorporated into the lipophilic regions of the niosomes, resulting in their enlargement.

The spherical shape of niosomes may be due to the nonionic surfactant used. In 2011, Israelachvili designed a geometric method known as the critical packing parameter, where its geometry can be determined through the characteristics of area and volume of the surfactant. This method uses Tandford equations for the calculation of nonpolar longitude, nonpolar group volume, and polar area, providing a dimensionless number. In the case of S60, it corresponds to 344.7, categorized as reverse micelles with a spherical structure. Reverse micelles are thermodynamically stable structures which minimize surface energy due to their shape, where the polar and nonpolar component’s structure compartments in the central nuclei of the reverse micelles allow dispersion [39,40].

## 3. Materials and Methods

### 3.1. Materials

Sorbitan monostearate (C_24_H_46_O) (CAS number 1338-41-6), cholesterol (C_27_H_46_O) (CAS number 57-88-5), dihexadecyl phosphate (DCP) [CH_3_ (CH_2_)_15_ O]_2_ (CAS number 2197-63-9), and triacontanol (C_30_H_62_O) (CAS number 593-50-0) were purchased from Sigma Aldrich Lab & Production Materials (Toluca, México). The rest of the reagents used in this work are of analytical grade unless stated otherwise.

### 3.2. Experimental Design

A Box–Behnken experimental design (3 variables and 3 different levels) was used to study the effect of independent variables on niosomes (Table 3). Three different variables were included: the cholesterol–Span 60 molar ratio, the amount of dihexadecyl phosphate, and sonication time. All response variables were analyzed by Design expert software, version 22 (Satatease Inc., Minneapolis, MN, USA).

### 3.3. Methods

The characterization of niosomes prepared by thin film hydration–ultrasonication was performed using Fourier-transform infrared spectroscopy, laser scanning confocal microscopy, scanning electron microscopy, and transmission electron microscopy, providing information about the shape, surface, size, stability, and molecular interactions of the prepared niosomes.

#### 3.3.1. Synthesis

Niosomes were prepared using the thin-film hydration method in combination with ultrasound [41]. Niosome components were added to a round-bottom flask at the molarities shown in Table 4 using 10 mL of chloroform and 1 mg of triacontanol, and manually stirred for 1 min. This mixture was taken to a rotary evaporator at 55 °C, 68 RPM, and −0.5 mbar for 30 min to obtain a thin film. The formed film was hydrated using 10 mL of ultrapure water at pH 7 and subjected to two agitation cycles. The first cycle was performed at 60 °C, 320 RPM, for 30 min, and the second cycle was carried out at room temperature with continuous agitation for 24 h. The synthesized niosomes were subjected to ultrasound using an Up200ST sonicator from Hielscher with 40 W, 70% amplitude, and a 100% pulse at the times shown in Table 4. The synthesized niosomes were centrifuged at 13,500 RPM at 4 °C for 30 min, and the pellet formed contained the niosomes with triacontanol encapsulated. The obtained niosomes were stored in refrigeration at 4 °C.

#### 3.3.2. Size and Polydispersity Analysis by Dynamic Light Scattering

The size of niosomes was determined using a Zetasizer 3600 Nano series from Malvern Instruments (Malvern (Worcestershire), UK), with a 4 mW He-Ne laser with a wavelength of 633 nm and a detector with an angle of 173°. For this analysis, a polystyrene cell was used, and 900 μL of deionized water and 100 μL of the niosome sample were added. The data obtained were analyzed using the Zetasizer software 7.1.

#### 3.3.3. Zeta Potential of Niosomes

The measurement of zeta potential was performed in a special polystyrene cell for this parameter, adding 100 μL of niosomes and 900 μL of deionized water, and then analyzing the information using the Zetasizer software.

#### 3.3.4. Fourier-Transform Infrared Spectroscopy Analysis

FTIR was used to study the reagents comprising the niosomes and the interactions between them using a Fourier-transform infrared spectrometer (IRAffinity-1 from Shimadzu corporation).

In total, 1 mL of the niosome sample from the 15 formulations was placed in 1.5 mL Eppendorf tubes and frozen at −20 °C, followed by lyophilization for 8 h. After lyophilization, spectroscopy was performed in the mid-infrared range (4000–400 cm^−1^) with a resolution of 4 cm^−1^ and 120 scans employed in the ATR mode.

#### 3.3.5. Laser Scanning Confocal Microscopy Analysis

Confocal microscopy was used to study the hydrophobic and hydrophilic layers of the niosome formulations to indicate their presence or absence. For this purpose, staining with Nile Red was performed to locate and quantify lipid molecules through fluorescent emission with maximum excitation/emission levels at 552–636 nm when in non-polar environments. A 1:1 solution of Nile Red and acetone (1 mg/1 mL) was prepared, followed by a second solution using distilled water at a 1:10 ratio, and then filtered. Taking 100 μL of the sample, 20 μL of the Nile Red solution was added for observation under a confocal-multiphoton microscope LSM 710 NLO Carl Zeiss (Oberkochen (BW), Germany).

#### 3.3.6. Morphology Analysis by Scanning Electron Microscopy (SEM)

In total, 10 μL of niosome samples was deposited on carbon tape-coated SEM sample holders. These samples were stored in a −20 °C freezer and then lyophilized. After lyophilization, 50 μL of 25% glutaraldehyde was added to the samples. The sample was then placed in a JEOL Field Emission Scanning Electron Microscope (JSM-7800F) (Tokyo (Tokyo), Japan) and a low-voltage scan at 2 KeV with a magnification of ×20,000 was performed using the secondary and backscattered electron detectors.

#### 3.3.7. Transmission Electron Microscopy (TEM)

Niosomes were analyzed using a TEM microscope (JOEL-200CX) with a 100 k accelerating voltage. Niosome samples were prepared by using a negative stain technique (2% uranyl acetate in water).

## 4. Conclusions

An optimization was carried out through the Box–Behnken experimental design using three independent variables (cholesterol and S60 ratio; amount of dihexadecyl phosphate and sonication time) at three different levels. A total of 15 formulations were obtained. ANOVA analysis indicated that sonication time is a determining factor in the size of niosomes; otherwise, the ratio of cholesterol–S60 and the amount of dihexadecyl phosphate seems not affect the size, polydispersity, and zeta potential of niosomes. Niosomes with nanometric size and an adequate zeta potential were obtained in formulation 14. This was revealed by scanning and transmission electron microscopy in addition to dynamic light scattering analysis.

Synthesized niosomes have potential to encapsulate high-hydrophobicity molecules like triacontanol based on the experiments performed by laser scanning confocal microscopy, due to Nile Red fluorescence, and FTIR spectroscopy, which reveals a typical signal of niosomes belonging to an intermolecular interaction between cholesterol and S60.

## Figures and Tables

**Table 1:** Formulation of niosomes. Optimum target of response; particle size (139.54–550.34); PDI (0.124–0.502); and zeta potential (−40.96–−23.72 mV).

Run	A: Span 60: Cholesterol	B: Dihexadecyl Phosphate	C: Sonication Time	Particle Size (nm)	Polydispersity	Zeta Potential (mV)
1	0	1	1	215.96 ± 1.17	0.198 ± 0.008	−30.04 ± 1.32
2	1	−1	0	218.96 ± 13.19	0.212 ± 0.008	−28.63 ± 2.13
3	1	−1	−1	455.24 ± 3.09	0.218 ± 0.019	−30.44 ± 2.11
4	0	1	1	232.2 ± 5.18	0.342 ± 0.017	−40.96 ± 1.36
5	1	0	1	283.52 ± 18.66	0.502 ± 0.303	−27.73 ± 1.12
6	1	0	−1	550.34 ± 6.68	0.256 ± 0.087	−28.42 ± 0.97
7	−1	−1	0	214.56 ± 1.29	0.143 ± 0.045	−32.76 ± 2.14
8	0	0	0	230.58 ± 1.29	0.372 ± 0.007	−35.9 ± 1.17
9	−1	1	1	246.96 ± 2.16	0.408 ± 0.013	−38.18 ± 2.69
10	−1	1	−1	414.28 ± 12.9	0.278 ± 0.093	−29.8 ± 0.77
11	0	1	−1	406.78 ± 6.00	0.124 ± 0.014	−32.54 ± 0.33
12	1	1	0	175.36 ± 1.71	0.198 ± 0.015	−27.2 ± 0.21
13	0	0	0	218.46 ± 2.49	0.198 ± 0.013	−23.72 ± 1.99
14	0	−1	1	139.54 ± 0.93	0.198 ± 0.008	−31.28 ± 1.21
15	−1	1	0	205.86 ± 7.23	0.448 ± 0.003	−34.9 ± 1.08

**Table 2:** Main assignments of wavenumbers present in niosome components.

Component	Wavenumber (cm^−1^)	Assignment
Span 60	3400	O-H
	1120	R-O-R
	1100	C-O
Cholesterol	3400	O-H
	3100	C=C
Dihexadecyl phosphate	1050	PO_4_^3^
Triacontanol	3400	O-H
	3200	C-O

**Table 3:** Box–Behnken design: dependent and independent variables of niosome formulations.

Factor		Low (−1)	Medium (0)	High (1)
	Independent variables			
A	Span 60–cholesterol (mM)	5.7	11.4	17.1
B	Dihexadecyl phosphate (mM)	0.6	1.2	1.8
C	Sonication time (minutes)	1	3	5
	Dependent variables			
A	Particle size			
B	Zeta potential			
C	Polydispersity			

**Table 4:** Formulation of niosomes.

Organic Phase								Aqueous Phase
Formulation		Span 60	Cholesterol	DCP	Sonication (min)	Triacontanol	Chloroform (mL)	Ultrapure Water (mL)
F1	mM	11.4	11.4	1.2	3	0.22	10	10
F2	mM	17.1	17.1	0.6	3	0.22	10	10
F3	mM	11.4	11.4	0.6	1	0.22	10	10
F4	mM	11.4	11.4	0.6	5	0.22	10	10
F5	mM	17.1	17.1	1.8	5	0.22	10	10
F6	mM	17.1	17.1	1.2	1	0.22	10	10
F7	mM	5.7	5.7	0.6	3	0.22	10	10
F8	mM	11.4	11.4	1.2	3	0.22	10	10
F9	mM	5.7	5.7	1.2	5	0.22	10	10
F10	mM	5.7	5.7	1.2	1	0.22	10	10
F11	mM	11.4	11.4	1.8	1	0.22	10	10
F12	mM	17.1	17.1	1.8	3	0.22	10	10
F13	mM	11.4	11.4	1.2	3	0.22	10	10
F14	mM	11.4	11.4	0.6	5	0.22	10	10
F15	mM	5.7	5.7	1.8	3	0.22	10	10

## Data Availability

All data are available in the manuscript.
